# A critical appraisal of guidelines for the management of knee osteoarthritis using Appraisal of Guidelines Research and Evaluation criteria

**DOI:** 10.1186/ar2339

**Published:** 2007-12-06

**Authors:** Stéphane Poitras, Jérôme Avouac, Michel Rossignol, Bernard Avouac, Christine Cedraschi, Margareta Nordin, Chantal Rousseaux, Sylvie Rozenberg, Bernard Savarieau, Philippe Thoumie, Jean-Pierre Valat, Éric Vignon, Pascal Hilliquin

**Affiliations:** 1 Département d'épidémiologie, biostatistiques et de santé au travail, Université McGill, Montréal, Canada; 2 Service de rhumatologie, hôpital Henri Mondor, Créteil, France; 3 Division of General Medical Rehabilitation, Geneva University Hospitals, Switzerland; 4 Department of Orthopaedics, New York University, New York, USA; 5 Agence Nukleus, Paris, France; 6 Département de rhumatologie, hôpital Pitié-Salpetrière, Paris, France; 7 Fédération de Médecine Physique et de Réadaptation, Hopital Rothschild APHP, Paris, France; 8 Université François-Rabelais de Tours, Faculté de Médecine, France; 9 Université Claude Bernard, Lyon, France; 10 Service de Rhumatologie, Centre Hospitalier Sud Francilien, Corbeil Essonnes, France

## Abstract

Clinical practice guidelines have been elaborated to summarize evidence related to the management of knee osteoarthritis and to facilitate uptake of evidence-based knowledge by clinicians. The objectives of the present review were summarizing the recommendations of existing guidelines on knee osteoarthritis, and assessing the quality of the guidelines using a standardized and validated instrument – the Appraisal of Guidelines Research and Evaluation (AGREE) tool. Internet medical literature databases from 2001 to 2006 were searched for guidelines, with six guidelines being identified. Thirteen clinician researchers participated in the review. Each reviewer was trained in the AGREE instrument. The guidelines were distributed to four groups of three or four reviewers, each group reviewing one guideline with the exception of one group that reviewed two guidelines. One independent evaluator reviewed all guidelines. All guidelines effectively addressed only a minority of AGREE domains. Clarity/presentation was effectively addressed in three out of six guidelines, scope/purpose and rigour of development in two guidelines, editorial independence in one guideline, and stakeholder involvement and applicability in none. The clinical management recommendation tended to be similar among guidelines, although interventions addressed varied. Acetaminophen was recommended for initial pain treatment, combined with exercise and education. Nonsteroidal anti-inflammatory drugs were recommended if acetaminophen failed to control pain, but cautiously because of gastrointestinal risks. Surgery was recommended in the presence of persistent pain and disability. Education and activity management interventions were superficially addressed in most guidelines. Guideline creators should use the AGREE criteria when developing guidelines. Innovative and effective methods of knowledge translation to health professionals are needed.

## Introduction

Osteoarthritis of the knee affects an important part of the population, causing disability in many individuals and engendering significant costs [1]. Its prevalence is also increasing, due in part to the aging of the population [2] and to higher obesity rates [3]. Clinical practice guidelines in the management of osteoarthritis of the knee have been elaborated to summarize evidence related to the management of this health problem and to facilitate uptake of evidence-based knowledge by clinicians. There has, however, been increased scrutiny of the quality of guidelines in recent years. This emphasis is in part related to the relatively recent work of the Appraisal of Guidelines Research and Evaluation (AGREE) collaboration, an 'international collaboration of researchers and policy makers working together to improve the quality and effectiveness of clinical practice guidelines by establishing a shared framework for their development, reporting and assessment' [4]. A review of the quality of knee osteoarthritis guidelines using the AGREE instrument was published in 2002, concluding that the quality of the guidelines varied and could generally be improved [5]. Several guidelines have been published or updated since then, following the advancements in knowledge regarding the management of this condition, particularly as it relates to nonsteroidal anti-inflammatory drugs (NSAIDs) and their cardiovascular safety.

The present review had the following objectives: to summarize the recommendations of existing guidelines on knee osteoarthritis; and to assess the quality of the guidelines using the AGREE criteria.

## Methods

The following databases were searched in order to find relevant guidelines: Medline, Embase and National Guideline Clearinghouse (guidelines.gov). The search strategy used was osteoarthritis and guideline(s) in the title and/or abstract and/or MESH heading. For selection, the guidelines had to meet the following criteria: published or updated between 2001 and August 2006, major focus on knee osteoarthritis, addressing the treatment of the condition, published in English or French, and available electronically.

Six guidelines were identified using this search strategy [6-11]. One guideline was a partial update [7] of a previously published guideline [12], and both of these were combined for the evaluation. Four of the guidelines were complete updates of previously published guidelines [6,8-10], while one guideline was entirely new [11]. The quality of prior versions of four of the guidelines [6-9] had been assessed in a previous review [5].

These guidelines were distributed to four groups of three or four evaluators. Each group reviewed one guideline, with the exception of one group that reviewed two guidelines. One independent evaluator reviewed all guidelines. In total, 13 clinician researchers (five rheumatologists, three physiotherapists, one physiatrist, one occupational health physician, one psychologist, one family physician, one physician specialized in medical information) participated in the review. In addition to the guidelines, each evaluator was asked to read the AGREE instrument training manual [4] and received a 2-hour training session. This AGREE tool was used to assess the quality of the guidelines and has been shown generally reliable [13,14].

The AGREE instrument is composed of 23 items organized into six domains: scope/purpose, stakeholder involvement, rigour of development, clarity/presentation, applicability, and editorial independence. Guidelines with a clear scope/purpose specifically describe objectives and patient applicability. Stakeholder involvement is successfully addressed when all relevant groups, including patients, are included in the guideline development process, with target users defined and guidelines piloted among them. Guidelines with rigour in their development use systematic methods to search and select evidence, with an explicit link between evidence and recommendation formulation. In guidelines effectively addressing clarity/presentation, specific and unambiguous key recommendations and management options are easily identifiable. Applicability involves discussing cost and organizational implications of the guideline, and providing monitoring tools. Editorial independence is effectively addressed when conflicts of interest and independence from funding bodies are clearly stated.

A domain score is calculated by adding the scores of the items in a domain and by standardizing the total out of 100%. Domain scores greater than 60% are considered effectively addressed, a cutoff value used in the AGREE instrument for overall assessment [4]. The guideline is strongly recommended if it rates high (three or four out of four) on the majority of items and most domain scores are above 60%, is recommended if it rates high (three or four) or low (one or two) on a similar number of items and most domain scores are between 30% and 60%, and is not recommended if it rates low (one or two) on the majority of items and most domain scores are below 30% [4].

Each evaluator independently reviewed the guideline that was assigned to their group, using the AGREE instrument. Each group then met on two separate occasions with electronic and telephone exchanges between the meetings. At the last meeting, disagreements on ratings of the individual items were discussed until a consensus was reached on all items.

## Results

### Description of guidelines

Table 1 presents the interventions and the time period covered by the guidelines. Medication and exercises were covered by almost all guidelines; injections, surgery, education and equipment by most guidelines; with other interventions (supplements and passive treatments) covered by the minority. One guideline exclusively focused on exercise, while another focused only on NSAIDs. Most guidelines graded their recommendations according to the strength of evidence [6,8,10,11], while one guideline graded only some recommendations [9] and another guideline graded none [7]. The grading of criteria, however, varied among guidelines (Table 2).

**Table 1:** Characteristics of the selected guidelines

Guideline	Intervention								
	
	Medication	Exercise	Surgery	Injections	Equipment	Education	Supplements	Passive treatments	Period covered with literature review
Canadian Consensus Conference [8]	X								2000–2004
Ottawa Panel [11]		X							Not mentioned (published in 2005)
Schnitzer/American College of Rheumatology [7]	X	X	X	X	X	X			Not mentioned (published in 2002)
European League Against Rheumatism [6]	X	X	X	X	X	X	X		1966–2002
Institute for Clinical Systems Improvement [9]	X	X		X	X	X	X	X	Not mentioned (published in 2004)
American Academy of Orthopaedic Surgeons [10]	X	X	X	X	X	X			1990–2000

**Table 2:** Criteria for recommendation grading

Ottawa Panel [11]	
A	Evidence from one or more randomized controlled trials of a statistically significant, clinically important benefit (>15%)
B	Statistically significant, clinically important benefit (>15%) if the evidence is from observational studies or controlled clinical trials
C+	Clinical importance (>15%) but no statistical significance
C	No clinically important difference and no statistical significance
D	Evidence from one or more randomized controlled trials of a statistically significant benefit favouring the control group
Canadian Consensus Conference [8] and European League Against Rheumatism [6]	
A	Meta-analysis of randomized controlled trial or at least one randomized controlled trial
B	At least one controlled study without randomization or at least one quasi-experimental study
C	Descriptive studies, such as comparative, correlation or case–control studies
D	Expert committee reports or opinions and/or clinical experience of respected authorities
American Academy of Orthopaedic Surgeons [10]	
A	Meta-analysis of multiple, well-designed controlled studies; or high-power randomized, controlled clinical trial; or consistent findings from multiple well-designed experimental studies; or low-power randomized, controlled clinical trials; or nonexperimental studies such as nonrandomized, controlled single-group, pre–post, cohort, time, or matched case–control series; or nonexperimental studies, such as comparative and correlational descriptive and case studies
B	Generally consistent findings from well-designed experimental studies; or low-power randomized, controlled clinical trials; or nonexperimental studies such as nonrandomized, controlled single-group, pre–post, cohort, time, or matched case–control series; or nonexperimental studies, such as comparative and correlational descriptive and case studies
C	Inconsistent findings from well-designed experimental studies; or low-power randomized, controlled clinical trials; or nonexperimental studies such as nonrandomized, controlled single-group, pre–post, cohort, time, or matched case–control series; or nonexperimental studies, such as comparative and correlational descriptive and case studies
D	Little or no systematic empirical evidence
Institute for Clinical Systems Improvement [9]	
1	Strong design study results that are clinically important and consistent. The results are free of any significant doubts about generalizability, bias, and flaws in research design. Studies with negative results have sufficiently large samples to have adequate statistical power
2	Strong design study results that are inconsistent or with minor doubts about generalizability, bias, flaws in research design, or adequacy of sample size. Alternatively, evidence consists solely of consistent results from weaker designs
3	Strong design study results that are substantially inconsistent or with serious doubts about generalizability, bias, flaws in research design, or adequacy of sample size. Alternatively, evidence consists solely of limited results from weaker designs

### AGREE evaluation of guidelines

In general, there were few disagreements among reviewers on AGREE scores, and all disagreements were resolved after discussion. Table 3 presents the item scores using the AGREE instrument, and Table 4 presents the domain scores and overall assessment of the guidelines. Only a minority of domains were effectively addressed by the guidelines. The Canadian Consensus Conference (CCC) guideline [8], the European League Against Rheumatism (EULAR) guideline [6], the Institute for Clinical Systems Improvement (ICSI) guideline [9] and the Ottawa Panel guideline [11] effectively addressed two domains, and the American Academy of Orthopaedic Surgeons (AAOS) guideline [10] and the Schnitzer/American College of Rheumatology (ACR) guideline [7] effectively addressed none. There was variability among guidelines in the domains effectively addressed.

**Table 3:** Appraisal of Guidelines Research and Evaluation of the guidelines

Appraisal of Guidelines Research and Evaluation criterion	EULAR [6]	Ottawa Panel [11]	ICSI [9]	CCC [8]	AAOS [10]	Schnitzer/ACR [7]
Scope/purpose						
1. Overall objective(s) specifically described	2	3	2	3	3	3
2. Clinical question(s) specifically described	3	2	4	2	2	3
3. Patients to whom the guideline is meant to apply specifically described	3	4	4	2	3	2
Stakeholder involvement						
4. Development group included individuals from all relevant professional groups	2	4	2	4	1	1
5. Patients' views and preferences sought	1	2	1	4	1	2
6. Target users clearly defined	1	4	4	1	4	1
7. Guideline piloted among end users	1	1	1	1	1	1
Rigour of development						
8. Systematic methods used to search for evidence	3	3	1	3	2	1
9. Criteria for selecting evidence clearly described	4	4	1	2	1	2
10. Methods used for formulating the recommendations clearly described	3	2	1	1	3	1
11. Health benefits, side effects and risks considered in formulating the recommendations	4	3	2	4	2	4
12. Explicit link between recommendations and supporting evidence	4	4	3	4	2	3
13. Guideline externally reviewed by experts prior to publication	1	3	1	1	2	1
14. Procedure for updating the guideline provided	1	1	3	3	2	1
Clarity/presentation						
15. Specific and unambiguous recommendations	3	2	3	2	2	1
16. Different options for diagnosis and/or treatment of the condition clearly presented	3	4	3	4	2	3
17. Key recommendations easily identifiable	4	3	4	4	4	1
18. Guideline supported with tools for application	2	2	4	2	2	1
Applicability						
19. Potential organizational barriers in applying the recommendations discussed	1	2	2	1	1	1
20. Potential cost implications of applying the recommendations considered	1	1	1	4	1	2
21. Guideline presents key review criteria for monitoring and audit purposes	1	1	3	1	1	1
Editorial independence						
22. Guideline editorially independent from the funding body	3	3	1	4	1	1
23. Conflicts of interest of guideline development members recorded	1	1	4	4	1	4

AAOS, American Academy of Orthopaedic Surgeons; ACR, American College of Rheumatology; CCC, Canadian Consensus Conference; EULAR, European League Against Rheumatism; ICSI, Institute for Clinical Systems Improvement. 1, Strongly disagree; 2, disagree; 3, agree; 4, strongly agree.

**Table 4:** Domain scores and overall assessment of the guidelines

Appraisal of Guidelines Research and Evaluation domain	EULAR [6]	Ottawa Panel [11]	ICSI [9]	CCC [8]	AAOS [10]	Schnitzer/ACR [7]
Scope/purpose (%)	56	67	78	44	56	56
Stakeholder involvement (%)	8	58	33	50	25	8
Rigour of development (%)	62	62	24	52	33	29
Clarity/presentation (%)	67	58	83	67	50	17
Applicability (%)	0	11	33	33	0	11
Editorial independence (%)	33	33	50	100	0	50
Overall quality assessment of the guideline	Recommended	Recommended	Recommended	Recommended	Not recommended	Not recommended

AAOS, American Academy of Orthopaedic Surgeons; ACR, American College of Rheumatology; CCC, Canadian Consensus Conference; EULAR, European League Against Rheumatism; ICSI, Institute for Clinical Systems Improvement.

The Ottawa Panel guideline and the CCC guideline can be considered to have the highest quality among the guidelines, since they effectively addressed two domains and came close to effectively addressing two others (≥ 50%). The Ottawa Panel guideline effectively addressed scope/purpose and rigour of development, but poorly addressed applicability and editorial independence. The CCC guideline effectively addressed clarity/presentation and editorial independence, but poorly addressed scope/purpose and applicability.

Next in quality would be the EULAR and ICSI guidelines, both effectively addressing two domains and coming close to addressing another one. The EULAR guideline effectively addressed rigour of development and clarity/presentation, but poorly addressed stakeholder involvement, applicability and editorial independence. The ICSI guideline effectively addressed scope/purpose and clarity/presentation, but poorly addressed stakeholder involvement, rigour of development, and applicability.

Finally, both the AAOS and the Schnitzer/ACR guidelines only came close to effectively addressing two domains. The AAOS guideline poorly addressed stakeholder involvement, rigour of development, applicability, and editorial independence. The Schnitzer/ACR guideline poorly addressed stakeholder involvement, rigour of development, clarity/presentation, and applicability.

On the basis of these scores, none of the guidelines were strongly recommended. The Ottawa Panel guideline, the CCC guideline, the EULAR guideline and the ICSI guideline were recommended, while the AAOS guideline and the Schnitzer/ACR guideline were not.

Clarity/presentation was the domain most often effectively addressed by the guidelines (three out of six guidelines), followed by scope/purpose and rigour of development (two out of six guidelines). Editorial independence was effectively addressed in only one guideline. The most poorly addressed domains were stakeholder involvement and applicability, with no guideline effectively addressing these.

### Guideline recommendations

Tables 5, 6, 7, 8, 9, 10, 11, 12 summarize the recommendations of the guidelines according to the intervention category. There was variability among guidelines in the specificity of the interventions studied, with some being more general and other guidelines more detailed. Only one guideline systematically provided recommendations according to the type of outcome pursued [11].

**Table 5:** Guideline recommendations for exercises

	Ottawa Panel [11]	AAOS [10]	EULAR [6]	ICSI [9]	Schnitzer/ACR [7]
Exercises			Recommended (A)		
Lower limb strengthening exercises	Recommended (A, C+ or C depending on type and outcome)	Recommended (A)		Recommended (1)	Recommended
Walking	Recommended (A, C+ or C depending on outcome)			Recommended (1)	
Whole-body exercises or physical activity	Recommended (A or C depending on outcome)			Recommended (1)	
Jogging in water	Recommended (A or C depending on outcome)				
Combined lower limb strengthening, flexibility and mobility exercises	Recommended (A or C depending on outcome)				
Aerobic exercises		Recommended (A)		Recommended (1)	Recommended
Lower limb range of motion or mobility or flexibility exercises		Recommended (A)		Recommended (1)	Recommended
Manual therapy with exercises	Recommended (A or C depending on outcome)				

AAOS, American Academy of Orthopaedic Surgeons; ACR, American College of Rheumatology; EULAR, European League Against Rheumatism; ICSI, Institute for Clinical Systems Improvement.

**Table 6:** Guideline recommendations for medication

	AAOS [10]	EULAR [6]	ICSI [9]	Schnitzer/ACR [7]	CCC [8]
Nonselective NSAID	Recommended (A)	Recommended (A)	Recommended if acetaminophen not effective	Recommended	Recommended (A)
		Recommended with PPI if gastrointestinal risk factors		Recommended with PPI or misoprol if gastrointestinal risk factors	Recommended (A) with PPI if gastrointestinal risk factors
				Use with caution for patients with high risk factors for congestive heart failure or renal problems	Use with caution with elderly patients (C) or patients with renal problems (D)
				Not recommended for patients on anticoagulation therapy or preoperative period	
Topical NSAID		Recommended (A)			Recommended (A)
Acetaminophen	Recommended (A)	Recommended (A) as initial pain treatment	Recommended as initial pain treatment	Recommended as initial pain treatment	Recommended (A) as initial pain treatment
Coxibs	Recommended (B) if renal or gastrointestinal risk factors	Recommended (A) if gastrointestinal risk factors	Recommended if gastrointestinal risk factors	Recommended for patients not responding to acetaminophen or nonpharmacologic modalities	Recommended (A) if gastrointestinal risk factors, depending on cardiovascular risks
				Recommended for patients with severe pain or signs of inflammation	Use with caution with elderly patients (C) or patients with renal problems (D)
				Recommended for patients with high gastrointestinal risks.	
				Use with caution for patients with high risk factors for congestive heart failure or renal problems	
Tramadol				Recommended for patients with contraindication to NSAIDs/coxibs or who have not responded to oral therapy	
Opioids		Recommended (B) if NSAIDs are contraindicated	Recommended if NSAIDs contraindicated and if nonpharmacologic treatments not effective	Recommended for patients who have not responded to tramadol or have side effects	
Capsaicin		Recommended (A)		Recommended as an adjunct treatment to oral therapy, for patients with contraindication to NSAIDs/coxibs or for patients who have not responded to oral therapy	
Nonacetylated salicylates				Recommended	
Methylsalicylate				Recommended as an adjunct treatment to oral therapy, for patients with contraindication to NSAIDs/coxibs or for patients who have not responded to oral therapy	

AAOS, American Academy of Orthopaedic Surgeons; ACR, American College of Rheumatology; CCC, Canadian Consensus Conference; EULAR, European League Against Rheumatism; ICSI, Institute for Clinical Systems Improvement; NSAID, nonsteroidal anti-inflammatory drug; PPI, proton pump inhibitor.

**Table 7:** Guideline recommendations for symptomatic slow-acting drugs

	EULAR [6]	ICSI [9]
Glucosamine	Recommended (A)	Recommended
Chondroitin	Recommended (A)	Recommended
Avocado/soya unsaponifiables	Recommended (B)	
Diacerein	Recommended (B)	

EULAR, European League Against Rheumatism; ICSI, Institute for Clinical Systems Improvement.

**Table 8:** Guideline recommendations for intraarticular injections

	AAOS [10]	EULAR [6]	ICSI [9]	Schnitzer/ACR [7]
Corticosteroid	Recommended (D) if inflammation	Recommended (B)	Recommended	Recommended as adjunct treatment to oral therapy, for patients with contraindication to NSAIDs/coxibs or for patients who have not responded to oral therapy
Hyaluronic acid		Recommended (B)	Recommended (2)	Recommended as adjunct treatment to oral therapy, for patients with contraindication to NSAIDs/coxibs or for patients who have not responded to oral therapy

AAOS, American Academy of Orthopaedic Surgeons; ACR, American College of Rheumatology; EULAR, European League Against Rheumatism; ICSI, Institute for Clinical Systems Improvement; NSAID, nonsteroidal anti-inflammatory drug.

**Table 9:** Guideline recommendations for surgery

	AAOS [10]	Schnitzer/ACR [7]	EULAR [6]
Surgery	Recommended for patients with 12 weeks or more of pain not responding to conservative treatment	Recommended for patients with severe osteoarthritis limiting their activities of daily living and not responding to nonpharmacologic and pharmacologic treatments	Recommended (C) for patients with radiographic evidence of osteoarthritis, refractory pain and disability
Total knee replacement	Recommended (A) for patients with bi/tri compartmental arthritis if no response from conservative treatment		Recommended (C)
	Recommended (A) for patients with medial compartment arthritis not candidate for osteotomy or unicompartmental knee replacement		
	Recommended (A) for patients with lateral compartment arthritis not candidate for osteotomy		
	Recommended (B) for older patients if magnetic resonance imaging confirms avascular necrosis		
	Recommended (B) for older or less active patients with isolated patellofemoral arthritis		
	Recommended (D) if no response from conservative treatment and previous infection		
	Not recommended (D) if active infection		
Unicompartmental knee replacement	Recommended (B) for less active patients with medial compartment arthritis		Recommended (C)
	Recommended (C) for patients with lateral compartment arthritis not candidate for osteotomy		
Osteotomy	Recommended (A) for young, active patients with medial compartment arthritis and varus alignment if no response from conservative treatment		Recommended (C)
	Recommended (B) for young, active patients with lateral compartment arthritis		
Arthroscopy	Not recommended (A) if no mechanical symptoms		Recommended (C)
	Recommended (B) if degenerative arthritis and mechanical symptoms		
	Recommended (B) if gross malalignment/instability, cartilage remaining and localized symptoms		
Knee fusion	Recommended (D) if no response from conservative treatment and previous infection, or for young patients with a history of chronic infection		
Patellectomy	Recommended (D) for young, active patients with isolated patellofemoral arthritis		

AAOS, American Academy of Orthopaedic Surgeons; ACR, American College of Rheumatology; EULAR, European League Against Rheumatism.

**Table 10:** Guideline recommendations for passive treatments

	Institute for Clinical Systems Improvement [9]
Cold/heat	Recommended
Compression/elevation	Recommended
Massage	Recommended if heat/cold and medications are contraindicated or not effective
Transcutaneous electrical nerve stimulation (TENS)	Recommended if heat/cold and medications are contraindicated or not effective
Acupuncture	Recommended if heat/cold and medications are contraindicated or not effective

**Table 11:** Guideline recommendations for equipment

	AAOS [10]	EULAR [6]	ICSI [9]	Schnitzer/ACR [7]
Assistive devices for ambulation or activities of daily living	Recommended (B)	Recommended	Recommended	Recommended
Orthotic devices/braces/taping	Recommended (B)	Recommended (B)	Recommended if heat/cold and medications are contraindicated or not effective	Recommended
Appropriate footwear or insoles	Recommended (B)	Recommended (B)	Recommended if heat/cold and medications are contraindicated or not effective	Recommended

AAOS, American Academy of Orthopaedic Surgeons; ACR, American College of Rheumatology; EULAR, European League Against Rheumatism; ICSI, Institute for Clinical Systems Improvement.

**Table 12:** Guideline recommendations for education

	AAOS [10]	EULAR [6]	ICSI [9]	Schnitzer/ACR [7]
Education	Recommended (D)	Recommended (A)	Recommended	Recommended
Weight loss if obese	Recommended (B)	Recommended (B)	Recommended	Recommended
Activity management or joint protection	Recommended (B)		Recommended	Recommended
Social support	Recommended (B)			Recommended
Stress management/relaxation			Recommended	

AAOS, American Academy of Orthopaedic Surgeons; ACR, American College of Rheumatology; EULAR, European League Against Rheumatism; ICSI, Institute for Clinical Systems Improvement.

### Exercises

Exercise was recommended in all guidelines that studied this intervention (Table 5), with the specificity of recommendations ranging from very general [6] to very specific [11]. Generally, lower limb strengthening, mobility and flexibility exercises were recommended. Aerobic exercises and general physical activity were also recommended. For the guideline that provided recommendations according to outcome [11], exercise appeared to have a positive impact on pain and disability.

### Medication and supplements

Acetaminophen was recommended as initial pain treatment in all guidelines (Table 6). NSAIDs were also recommended, but combined with a proton pump inhibitor in the presence of high gastrointestinal risk factors. Alternatively, coxibs were also recommended. The cardiovascular safety of both NSAIDs and coxibs was questioned in one guideline [8]. Some guidelines recommended other drugs if the preceding medications were either contraindicated or were nonresponsive [6,7,9]. Symptomatic slow-acting drugs were recommended in certain guidelines: glucosamine and chondroitin were recommended in two guidelines [6,9], while avocado/soya unsaponifiables and diacerein were recommended in one guideline [6] (Table 7).

### Intraarticular injections

Corticosteroid or hyaluronic acid injections were recommended in four of the guidelines [6,7,9,10] (Table 8), but with less strength of evidence when compared with exercises or medication. The injections were mostly recommended as second-line treatments, with relatively short-term benefits for corticosteroids.

### Surgery

Three guidelines provided recommendations regarding surgery [6,7,10], with one providing detailed recommendations according to the type of intervention and the patients' condition [10] (Table 9). Surgery was generally recommended in chronic pain patients with moderate to severe disability for whom conservative treatment had not been effective or was insufficient.

### Passive treatments

Five adjunct treatments, consisting of heat/ice, compression/elevation, transcutaneous electrical nerve stimulation (TENS), massage and acupuncture, were recommended in one guideline [9] (Table 10). None of the other guidelines provide recommendations towards other passive treatments.

### Equipment

Three categories of equipment were recommended in four of the guidelines [6,7,9,10]: assistive devices for ambulation and activities of daily living, knee orthotics, and appropriate footwear (Table 11). Referring the patient to a health professional trained in the use of these equipments was generally recommended.

### Education

Education and weight loss was recommended in four guidelines [6,7,9,10] (Table 12), although the term 'education' was clearly defined in only one guideline [9]. Activity management, including activities of daily living, leisure, sports and work, was briefly addressed in three guidelines [7,9,10].

## Discussion

The present review highlights the relatively large number of types of interventions available to clinicians and patients when managing knee osteoarthritis. Types of interventions included in the guidelines varied, reflecting choices made by development teams. It appears that interventions with the strongest evidence tended to be addressed in most guidelines (such as exercise and medication), while other interventions with less evidence tended to be addressed in a minority of guidelines. There was also variability in the level of details of interventions, with some guidelines dividing a category of intervention into various forms, and others succinctly describing only the category. The interests, mandate and resources of the development team probably guided the type and extent of interventions addressed.

When comparing guidelines, there generally seemed to be agreement in recommendations on the interventions addressed. Acetaminophen was generally recommended for initial pain treatment. Introducing more potent medication, such as NSAIDs, was also generally suggested if acetaminophen failed to control pain. The gastrointestinal risks associated with NSAID intake was stressed in the guidelines, however, especially with patients with high gastrointestinal risk factors. Only the most recent guideline [8] discussed the cardiovascular safety of NSAIDs following the 2005 advice by the American Food and Drug Agency [15]. This seems to highlight the slowness of guidelines to react to important emerging data. This observation also shows that guidelines can rapidly become outdated, especially in fields of rapid knowledge advancements. For the guidelines included in the present review that were updates [6-10], there was a delay of 1–7 years between versions, with a mean of 3.8 years. These results are probably biased by the fact that most of the included guidelines were published in peer-reviewed journals, involving delays for publication. The two guidelines that were not published in peer-reviewed journals [9,10], however, had the shortest (1 year) and longest (7 years) delays between versions. Innovative knowledge translation methods, allowing the rapid integration of new evidence by clinicians, should be developed and implemented.

Exercise and education were also generally recommended throughout all disease stages. The type of exercise recommended varied among guidelines, but it appears the important notion is to keep active, whatever the type of exercise. Although education was frequently suggested, its elements were not well described in the guidelines, apart from one [9]. Perhaps this is related to the relative lack of evidence regarding the effectiveness of specific messages given to patients. Activity management was also not detailed in the guidelines, although knee osteoarthritis often has an important impact on the patient's functional capacities [1]. Referral to an occupational therapist was sometimes suggested to help in this management. Future guidelines should specify education and activity management interventions, in order to help in their application.

Surgery was generally recommended as a last resort in the presence of persistent pain and disability. Other interventions were suggested in some of the guidelines, such as intraarticular injections, supplements, equipment and passive therapies, but their role and place in the management of knee osteoarthritis was unclear. This is probably related to the weaker evidence regarding the effectiveness of these interventions. The role of these interventions should be specified in future guidelines.

### AGREE evaluation of guidelines

The AGREE evaluation demonstrated that the guidelines effectively addressed only a minority of domains. Although scope/purpose, rigour of development and clarity/presentation were the most often effectively addressed domains, the majority of guidelines failed to appropriately address these domains. Guideline developers should focus on the AGREE criteria constituting these domains in the elaboration of future guidelines.

Three domains were particularly not well addressed by the guidelines: stakeholder involvement, applicability, and editorial independence. In an AGREE evaluation of low-back pain guidelines, very similar results were obtained [16]. It therefore appears that guidelines in general have difficulty addressing these dimensions, and several hypotheses can be elaborated to explain this. Regarding editorial independence, this was often simply not mentioned in the guidelines. It is not possible to know whether this was an error of omission or whether there were conflicts of interest. Guideline developers should explicitly mention editorial links.

As for stakeholder involvement, it appears that a change in the attitude of guideline developers could be needed. Guideline developing teams tended not to include all relevant stakeholders and patients. It is, however, suggested that involving stakeholders in guideline elaboration tends to improve applicability of the recommendations and to facilitate appropriation among end users [17]. Although there is evidence describing ways to facilitate this collaboration [18,19], guideline developers are perhaps unaware of this literature or are uncomfortable in sharing power and responsibilities, especially with patients. Guideline developers should be made aware of the literature on stakeholder involvement and its advantages, and methods facilitating this collaboration should be developed and used.

As for guideline applicability, barriers in guideline use should be taken into account during guideline development, in order to facilitate use and uptake [20,21]. Expecting guideline developers to address comprehensively this domain while developing the guideline, however, is perhaps unrealistic. A more incremental approach to guideline development has been suggested [22], in which a guideline is elaborated with stakeholders taking into account potential barriers raised during the process. This is followed by piloting the guideline with end users and collecting organizational and financial barriers with monitoring instruments. Taking into account these results, a final version of the guideline is elaborated before general diffusion and implementation. The cost-effectiveness of such a process, however, remains to be demonstrated.

It appears that the AGREE criteria are more and more taken into account when elaborating guidelines. The two most recent guidelines [8,11] had the highest quality and were the only ones mentioning the use of the AGREE instrument in the elaboration. Even these guidelines, however, failed to effectively address the majority of domains. Producing a high-quality guideline effectively addressing all AGREE domains appears to remain a challenge.

## Conclusion

Therapeutic interventions addressed in the guidelines varied, with no guideline addressing all interventions. When an intervention was addressed in two or more guidelines, the corresponding clinical management recommendation tended to be similar among guidelines. Acetaminophen was recommended for initial pain treatment, combined with exercise and education. NSAIDs were recommended if acetaminophen failed to control pain, but cautiously because of gastrointestinal risks. Surgery was recommended in the presence of persistent pain and disability. Education and activity management interventions were superficially addressed in most guidelines and should be detailed in the future. Guidelines effectively addressed only a minority of AGREE domains. In order to improve applicability and to increase uptake by end users, stakeholder opinions and barriers in use need to be taken into account during guideline development. The apparent slowness of guideline development processes to integrate and disseminate new knowledge means that innovative methods of knowledge translation to health professionals should be developed.

## Abbreviations

AAOS = American Academy of Orthopaedic Surgeons; ACR = American College of Rheumatology; AGREE = Appraisal of Guidelines Research and Evaluation; CCC = Canadian Consensus Conference; EULAR = European League Against Rheumatism; ICSI = Institute for Clinical Systems Improvement; NSAID = nonsteroidal anti-inflammatory drug.

## Competing interests

The authors declare that they have no competing interests. This research was funded by Laboratoires Expanscience, Courbevoie, France, which manufactures one of the medications discussed in the present review (avocado/soya unsaponifiables). The views or interests of the funding body did not influence the content of the manuscript.

## Authors' contributions

All authors participated in conception and design of the study, in the acquisition and interpretation of data, and in the revision of the manuscript. All authors read and approved the final manuscript. SP additionally drafted the manuscript
